# Development and Application of Physiologically-Based Pharmacokinetic Model to Predict Systemic and Organ Exposure of Colorectal Cancer Drugs

**DOI:** 10.3390/pharmaceutics17010057

**Published:** 2025-01-03

**Authors:** Sara Peribañez-Dominguez, Zinnia Parra-Guillen, Iñaki F. Troconiz

**Affiliations:** 1 Department of Pharmaceutical Science, School of Pharmacy and Nutrition, University of Navarra, 31009 Pamplona, Spain; zparra@unav.es (Z.P.-G.); itroconiz@unav.es (I.F.T.); 2 Navarra Institute for Health Research (IdiSNA), 31002 Pamplona, Spain; 3 Navarra Institute of Data Science and Artificial Intelligence, DATAI, University of Navarra, 31009 Pamplona, Spain

**Keywords:** PBPK, physiologically-based pharmacokinetic model, colorectal cancer, CRC, pharmacokinetics, drug distribution, mechanistic modeling

## Abstract

Background/Objectives: Colorectal cancer (CRC) holds the third and second position among cancers affecting men and women, respectively. Frequently, the first-line treatment for metastatic CRC consists of the intravenous administration of 5-fluorouracil and leucovorin in combination with oxaliplatin or irinotecan. Physiologically-based pharmacokinetic models (PBPK) aim to mechanistically incorporate body physiology and drug physicochemical attributes, enabling the description of both systemic and organ drug exposure based on the treatment specificities. This bottom-up approach represents an opportunity to personalize treatment and minimize the therapeutic risk/benefit ratio through the understanding of drug distribution within colorectal tissue. This project has the goal of characterizing the systemic and tissue exposure of four anti-cancer drugs in humans using a PBPK platform fed with data from the literature. Methods: A literature search was performed to collect clinical data on systemic concentration versus time profiles. Physicochemical features were obtained from the literature, as well as parameters associated with distribution, metabolism, and excretion. The PBPK models were built using PK-Sim^®^. Results: The data from 51 clinical studies were extracted and combined in one single dataset. The PBPK models successfully described the exposure vs. time profiles with respect to both, with both the typical tendency and dispersion shown by the data. The percentage of observations falling within the two-fold error bounds ranged between 94 and 100%. The colon/plasma AUC_inf_ ratios were similar for 5-FU, oxaliplatin, and leucovorin, but it was significantly higher for irinotecan. Conclusions: The PBPK models support tailored treatment approaches by linking in vitro studies to organ exposure. These models serve as the initial step towards incorporating a dedicated tumor compartment, which will further account for the variability in tumor microenvironment characteristics to improve therapeutic strategies.

## 1. Introduction

Colorectal cancer ranks third among cancers impacting men and second among those affecting women, making it one of the most prevalent forms of cancer [1]. While complete surgical removal remains pivotal for curative treatment, enhanced oncological results have been attained through the incorporation of systemic neoadjuvant and adjuvant therapy [2]. Frequently, the first-line treatment for mCRC consists of the intravenous administration of 5-fluorouracil and leucovorin in combination with oxaliplatin or irinotecan, which are regimens that are commonly referred to as FOLFOX (fluorouracil, leucovorin, oxaliplatin) and FOLFIRI (fluorouracil, leucovorin, irinotecan) [3].

Although numerous studies have extensively detailed the pharmacokinetics of these drugs [4,5,6,7], there remains a notable gap in our understanding regarding their spreading within the organs of the body. Understanding the distribution of the parent drug is crucial, but it is also important to describe the pharmacokinetics of their active metabolites. Irinotecan, 5-FU, and leucovorin are metabolized into 7-ethyl-10-hydroxycamptothecin (SN-38), dihydrofluorouracil (FUH_2_), and 5,10-methylenetetrahydrofolate (folitixorin), respectively. In contrast, oxaliplatin does not undergo an enzymatic process to produce its active molecule. While the pharmacokinetics of SN-38, FUH_2_, and folitixorin have been studied [8], their behavior within physiological structures is less understood. Gaining insight into how anticancer drugs and their metabolites are distributed in the body is relevant for effective treatment. This holds particular significance, especially within organs embracing tumor growth, as they represent the primary target sites for drug action. Specifically concerning colorectal cancer, the comprehension of drug disposition relative to different segments of the colon, where tumors may be localized, is important for tailoring treatment to the characteristics of the cancer. In the context of colorectal cancer, understanding the disposition of drugs across various segments of the colon, where tumors may be localized, becomes of special interest. This is because the characteristics of the tumor—such as its size, location, and metabolic activity—can vary significantly across different regions of the colon. By comprehensively understanding how drugs are distributed in these specific areas, we can personalize treatment strategies based on the distinct features of the cancer. This personalized approach would help optimize therapeutic efficacy, minimize toxicity, and potentially improve patient outcomes by adapting drug delivery to the unique pharmacokinetic profile of the tumor in each individual.

Additionally, it is widely recognized that antitumoral therapies are required to find a balance between toxicity and effectiveness. Hence, understanding the dispersion of these drugs throughout the body facilitates the identification of regions where drug accumulation occurs and where potential side effects may manifest in patients.

Consequently, physiologically-based pharmacokinetic (PBPK) models represent a useful technique to study the biodistribution of these antitumoral drugs in the body. PBPK models aims to mechanistically incorporate body physiology and drug physicochemical attributes, enabling the description of both systemic and tissue drug exposure based on the treatment specificities [9]. This bottom-up approach not only helps us to comprehend the drug distribution, metabolism, and elimination in body organs, but also presents an opportunity to personalize treatment based on individual patient characteristics, thereby minimizing the therapeutic risk/benefit ratio. Moreover, PBPK models can be scaled to accommodate special populations such as elderly or pediatric patients. As a result, the development of PBPK models has expanded within the pharmaceutical industry, reaching a stage where the development of such models is recommended by regulatory agencies such as the European Medicines Agency (EMA) and the Food and Drug Administration (FDA) from United States [10].

Given the aforementioned considerations, the objective of this study is to build four PBPK models for irinotecan, 5-FU, oxaliplatin, and leucovorin to investigate the distribution of these antitumoral drugs and their active metabolites in colonic tissue, which represents the primary site of pharmacological action and potential toxicity.

## 2. Materials and Methods

### 2.1. Software

Data from the literature was scanned using WebplotDigitalizer tool. Datasets were built using R version 4.0.5 through RStudio interface version 1.4.1106. Model development was performed using PK-Sim^®^ (Open Systems Pharmacology Suite11) software.

### 2.2. Clinical Literature Data

**Figure 1:**
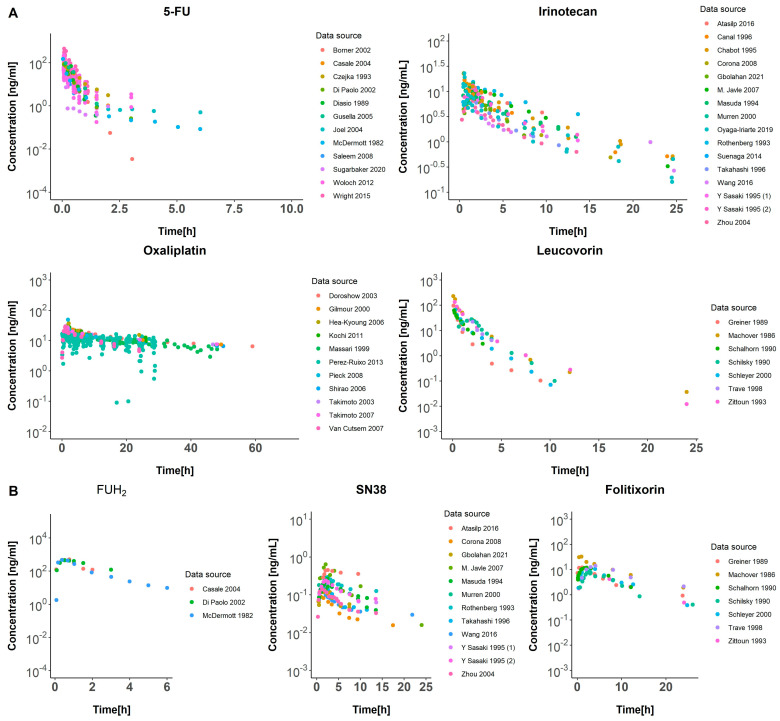
Dose normalized concentration vs. time profiles of main molecules (**A**) and active metabolites (**B**). Colors of dots represent various data sources: 5-FU sources: Borner 2002 [11], Casale 2004 [12], Czejka 1993 [13], Di Paolo 2002 [14], Diasio 1989 [7], Gusella 2005 [15], Joel 2004 [16], Mc Dermont 1982 [8], Saleem 2008 [17], Sugarbaker 2020 [18], Woloch 2012 [19], Wright 2015 [20]. Irinotecan soures: Atasilp 2016 [21], Canal 1996 [22], Catimel 1995 [23], Chabot 1995 [24], Corona 2008 [25], Gbolahan 2021 [26], M. Javle 2007 [27], Masuda 1994 [28], Murren 2000 [29], Rothenberg 1993 [30], Suenaga 2014 [31], Takahashi 1996 [32], Wang 2016 [33], Y Sasaki 1995 (1) [34], Y Sasaki 1995 (2) [35], Zhou 2004 [36]. Oxaliplatin sources: Doroshow 2003 [37], Gilmour 2000 [38], Hea-Kyoung 2006 [39], Kochi 2011 [40], Massari 1999 [41], Perez-Ruixo 2013 [42], Pieck 2008 [43], Shirao 2006 [44], Takimoto 2003 [45], Takimoto 2007 [46], Van Custem 2017 [47]. Leucovorin sources: Greiner 1989 [48], Machover 1986 [49], Schalhorn 1990 [50], Schilsky 1990 [51], Schleyer 2000 [52], Trave 1988 [53], Zittoun 1993 [6].

An extensive literature search was conducted to compile clinical studies for intravenous administration of irinotecan, oxaliplatin, 5-FU, and leucovorin. Articles were selected if at least a complete description of the dosing schedule was provided together with longitudinal exposure data, including individual profiles, average profiles, or a pull of data profiles. Relevant information included in the dataset consists of dose levels, dosing regimens, time of dosing and sampling, drug concentration levels, and covariates including age and gender. The same process was followed to obtain clinical data from the active metabolites of the molecules. Raw concentration profiles over time for each parent drug and their active metabolites are shown in Figure 1. Additional information on the sources and characteristics of the data used can be found in Table S1 of the Supplementary Materials.

### 2.3. PBPK Model Development

First, a literature search was performed to obtain drug-related parameters, including physicochemical characteristics and properties related with the distribution, metabolism, and elimination processes. For each drug studied, values for both characteristic types are listed in Table 1.

The four models were built in PK-Sim^®^ using standard small molecule model, which divides each organ into four subcompartments: blood cells, plasma, interstitial space, and cellular space. It incorporates a permeation barrier between plasma and interstitial space, accounting for the lower protein content in the interstitial space compared to plasma, and is well-suited for small molecules. Partitioning between plasma and interstitial space depends on protein concentration differences and the unbound plasma fraction, assuming equal affinity for plasma and interstitial proteins. Permeation through the endothelial barrier is determined by the product of endothelial permeability and surface area.

Briefly, and with respect to its elimination characteristics, irinotecan is metabolized to the active metabolite 7-ethyl-10-hydroxycamptothecin (SN-38) by carboxylesterase. It is also converted by CYP3A4 into two inactive metabolites: 7-ethyl-10-(4-amino-1-piperidino) carbonyloxycamptothecin (NPC) and 7-ethyl-10-[4-N-(5-aminopentanoic acid)-1-piperidino] carbonyloxycamptothecin (APC) [59]. In addition to its metabolism, irinotecan undergoes other renal and biliary excretion [55,59].

**Figure 2:**
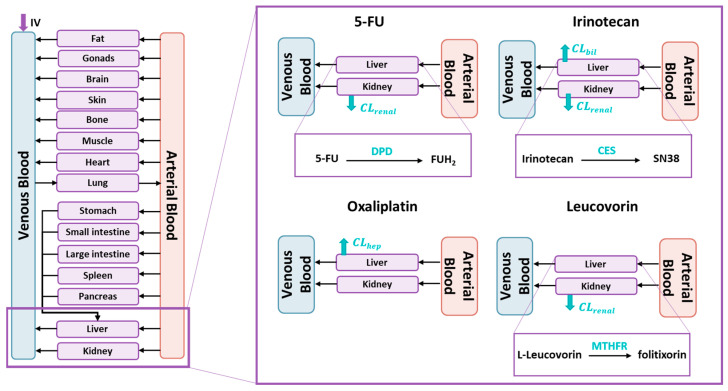
Schematic representation of four physiologically-based pharmacokinetic models. Models represent all organs connected by venous and arterial blood. Intravenous administration is represented as IV. Metabolism of the primary molecules is displayed in the box. CL_renal_, renal clearance; CL_bil_, biliary clearance; CL_hep_, total hepatic clearance; CES, carboxylesterase; SN38, 7-ethyl-10-hydroxycamptothecin; DPD, dihydropyrimidine dehydrogenase; FUH_2_, dihydrofluorouracil; and MTHFR, 5,10-methylenetetrahydrofolate reductase.

Most of the dose of 5-FU (90%) undergoes saturable catabolism by dihydropyrimidine dehydrogenase (DPD), leading to the formation of dihydrofluorouracil (FUH_2_), while the rest is excreted in the urine [60]. Oxaliplatin undergoes a non-enzymatic conversion to active derivatives and its elimination only involves the renal pathway [61]. Leucovorin is administered as a racemic mixture and only one of the isomers, L-leucovorin, shows pharmacological activity. Leucovorin is metabolized by 5,10-methylenetetrahydrofolate reductase, obtaining 5,10-methylenetetrahydrofolate (folitixorin). Additionally, leucovorin is primarily eliminated through renal excretion. [6]. Figure 2 shows the main PBPK model structure with the metabolic process schemes for each parent drug.

For the drugs metabolized by CYP enzymes, organ expression of these isoforms was included according to the expression profile from PK-Sim^®^ (Open Systems Pharmacology Suite 11) using the default database obtained through reverse transcription-polymerase chain reaction (RT-PCR).

### 2.4. Virtual Population

The system related parameters (e.g., organ volumes and flow rates) are provided by PK-Sim^®^. Furthermore, a population of one thousand patients (50% females) with ages between 25 and 80 and weights range of 37.5–130 kg was generated to perform a simulation for each drug. In addition, a second population, with the same characteristics as the previous one, was created including variability in elimination parameters. This variability was included as a coefficient of variation equal to 50% of the elimination parameters.

### 2.5. Model Evaluation

Model performance for each compound was first evaluated by visually exploring the agreement between the observed and model-based simulated plasma vs. time concentration profiles. Simulations were summarized by representing the 95% prediction intervals computed from the one thousand individual simulated profiles.

Numerical evaluations were also considered as criteria to evaluate performance of PBPK models according to previous PBPK model-based studies: (i) *FE* (fold error) within a two-fold range, (ii) *AFE*, the average fold error, and (iii) *AAFE*, the absolute average fold error (see Equations (1)–(3)) [62,63,64]:

(1) $$FE=\frac{predicted}{observed},$$

(2) $$AFE=10^{\frac{1}{n}\sum log\frac{predicted}{observed}},$$

(3) $$AAFE=10^{\frac{1}{n}\sum \left|\log\frac{predicted}{observed}\right|},$$

where *n* corresponds to the number of concentration data points. Predictions were categorized as satisfactory when AFE fell within the range of 0.8 to 1.25, and as acceptable when AFE ranged from 0.5 to less than 0.8, or from greater than 1.25 to 2. AFE values outside these ranges were deemed as poorly described. Similarly, the assessment of the AAFE followed a similar rationale, with values of 1.25 or lower considered satisfactory, values between 1.25 and 2 categorized as acceptable, and values exceeding 2 regarded as poor [65,66].

### 2.6. Model Exploration

Simulations were conducted to calculate the magnitude of drug exposure in plasma and each part of the colon (ascendens, descendens, transversum, and sigmoid), and were expressed as AUC_inf_ (µmol·min/L), representing the area under the concentration vs. time curve from time of administration until the drug is completely eliminated from the body. For each of the previously mentioned colon sections, AUC_inf_ was calculated in the vascular, interstitial, and intracellular compartments, as well as in venous plasma, to obtain a more detailed understanding of drug distribution across different target sites. The vascular AUC_inf_ was adjusted to account for the hematocrit, which represents 0.47 of the total blood volume, in accordance with PK-Sim standards. To calculate the total AUC_inf_ for each section of the colon, the contributions from the different compartments were considered, with fractions of 0.13, 0.14, and 0.73 for the vascular, intracellular, and interstitial spaces, respectively, based on PK-Sim standards. To facilitate comparison of AUC_inf_ between drugs, a standardized administration protocol of a 1 mg bolus was used for all simulations.

Additionally, as reported in previous studies on pharmacokinetic (PK) models of these drugs [67], certain covariates can influence the outcomes. The most important covariates identified in these earlier models are gender and body weight. Simulations were conducted to determine how covariates such as gender and body weight affect the concentration–time profiles in the target site, specifically the colon.

### 2.7. Model Assumptions and Limitations

One limitation of the model is the lack of data from the colon, primarily because it is uncommon to obtain samples from patients’ colons due to ethical considerations. This prevented us from verifying the model using data from this compartment; however, we conducted validation using plasma concentration data.

Additionally, our strategy was to use the PK-Sim standard model as a general approach for all drugs to maintain consistency in the estimation of Kp values across all compounds and tissues.

## 3. Results

### 3.1. Data Description

Data from 51 clinical studies, consisting of 16, 16, 11, and 8 studies for 5-fluorouracil, irinotecan, oxaliplatin, and leucovorin, respectively, were compiled into separate datasets for each drug. The datasets yielded a total of 410, 222, 392, and 48 observations across 27, 17, 11, and 7 concentration–time profiles for 5-fluorouracil, irinotecan, oxaliplatin, and leucovorin respectively.

Regarding metabolites, data from 22 clinical studies, consisting of 3, 12, and 7 for FUH_2_, SN38, and folitixorin respectively, were assembled into distinct datasets for each drug. The datasets yielded a total of 24, 212, and 188 observations across 3, 14, and 6 concentration–time profiles for FUH_2_, SN38, and folitixorin, respectively. These observations encompassed individual, mean, and pooled data profiles, as detailed in Supplementary Materials Table S1.

### 3.2. PBPK Model Development

**Figure 3:**
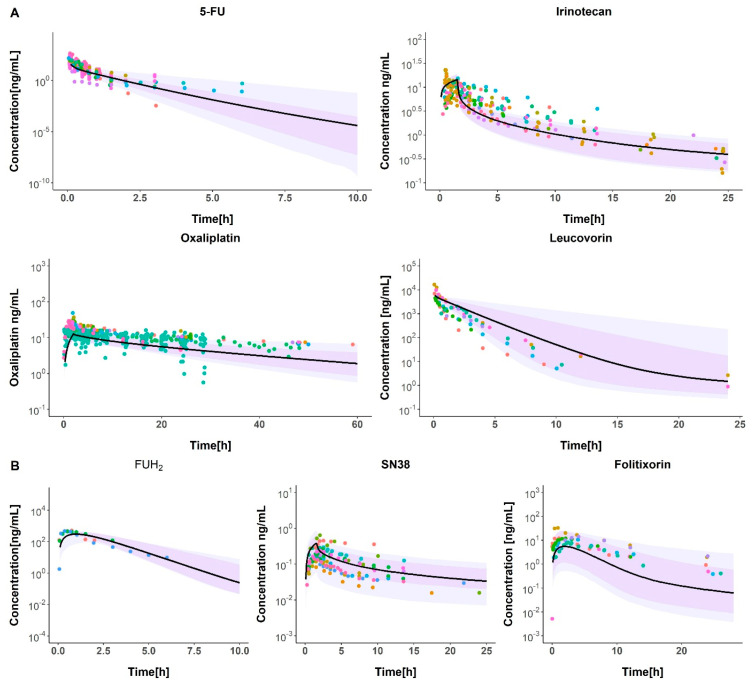
The model prediction for each drug vs. the observations over time. The solid black lines represent the model predictions and the colored points represent the training data observations. The areas cover the 95% prediction intervals around the 50th percentiles calculated for each of the 1000 simulated datasets. The darker region indicates the percentiles from dataset simulations accounting for variability in age and weight only, while the lighter region represents simulations that include variability in the elimination parameters as well. **Panel A** illustrates the primary molecules, while **Panel B** delineates the metabolites derived from these primary molecules.

As described in the Materials and Methods Section, the PBPK models were built considering drug-related parameters, system parameters, and population/individual characteristics, considering the known elimination mechanisms. Figure 3 shows the model predictions (solid black line) and observed profiles (colored dots) for the parent molecule and metabolites of each drug investigated, indicating proper model performance. The oxaliplatin model could not be expanded because this drug does not undergo a metabolic process to produce an active molecule. As a result, only predictions related to the three expanded models are shown in Figure 3. Both the parent drugs and their active metabolites described the data successfully.

### 3.3. Model Evaluation

Figure 3 illustrates the results derived from a simulation diagnostic assessment of the model, suggesting that the four models effectively represent both the typical pattern and the variability in observations across time. This variability is depicted by incorporating two distinct populations with different degrees of variability, represented by darker and lighter areas. Both the 5-FU and leucovorin models show a better description of the fluctuation of the observations, compared to irinotecan and oxaliplatin models that show some observations out of the range of the 5th and 95th percentiles in both areas, with 22 and 53 outliers for irinotecan and oxaliplatin, respectively.

**Figure 4:**
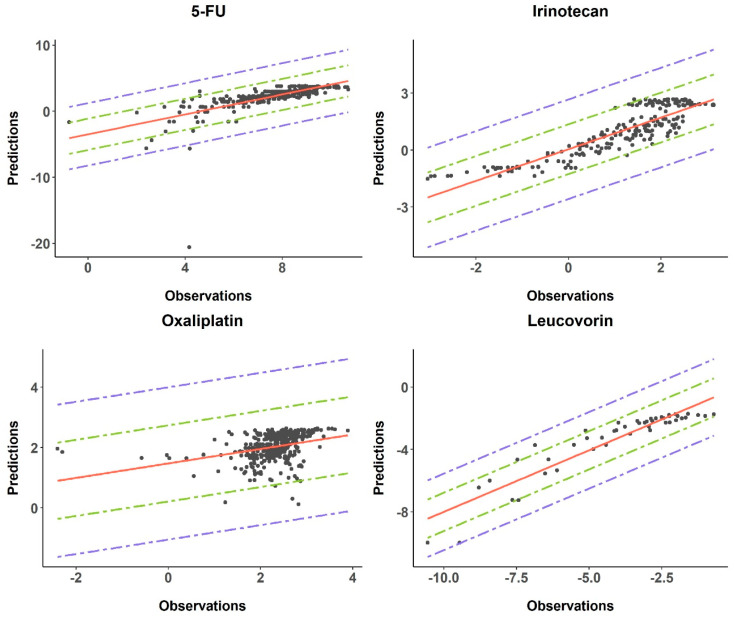
The goodness-of-fit plot for the four PBPK models of the predicted vs. observed concentrations. The solid red line represents the tendency line. The green and blue dashed lines represent the fold-error and 2-fold-error ranges.

Figure 4 displays the goodness-of-fit plots delineating the performance of the individual drug models. The percentage of observations falling within the two-fold error bounds, delineated by the blue dashed lines in the figure, are as follows: 100%, 94%, 100%, and 96% for irinotecan, 5-FU, oxaliplatin, and leucovorin, respectively. Additionally, the percentage of observations situated within the fold-error lines, drawn in green dashed lines in the figure, are as follows: 97%, 61%, 94%, and 72% for irinotecan, 5-FU, oxaliplatin, and leucovorin, respectively.

Moreover, a numerical validation via the determination of the AFE and AAFE values indicated satisfactory and/or acceptable predictive capabilities for AUC_0-tend_, C_max_, and AUC_inf_, with the exception of the AAFE value for the C_max_ for the 5-FU model, and the AFE and AAFE values for the AUC_inf_ for the oxaliplatin model (refer to Table 2 for details).

### 3.4. Model Exploration

The results of the model exploration process are summarized and shown in Table 3. The table includes AUC_inf_ values (µmol·min/L) for the ascending colon. The AUC_inf_ values for the other sections of the colon (descending colon, transverse colon, and sigmoid colon) were virtually identical to those of the ascending colon. Comparing the venous blood AUC_inf_, higher values are represented by the oxaliplatin profile, followed by the leucovorin, 5-FU, and irinotecan profiles.

Concerning the distinct compartments (vascular, interstitial, and intracellular), the AUC_inf_ varies across all drugs except for 5-FU, where notably similar values were observed in the vascular and interstitial spaces, with lower values in the intracellular space. Irinotecan and leucovorin demonstrate the highest AUC_inf_ in the intracellular compartment, compared to the rest of the drugs that show higher AUC_inf_ values in the interstitial (5-FU) or vascular (oxaliplatin) compartments.

With respect to the metabolites, when a bolus dose of 1 mg of the main molecule was given, the AUC_inf_ values were 5.57/5.94, 0.75/1.76, and 4.63/1.95 µmol × min/L in the plasma/colon for FUH_2_, SN38, and folitixorin, respectively, with SN38 being the only metabolite with a greater AUC_inf_ value in the colon compared to the plasma.

**Figure 5:**
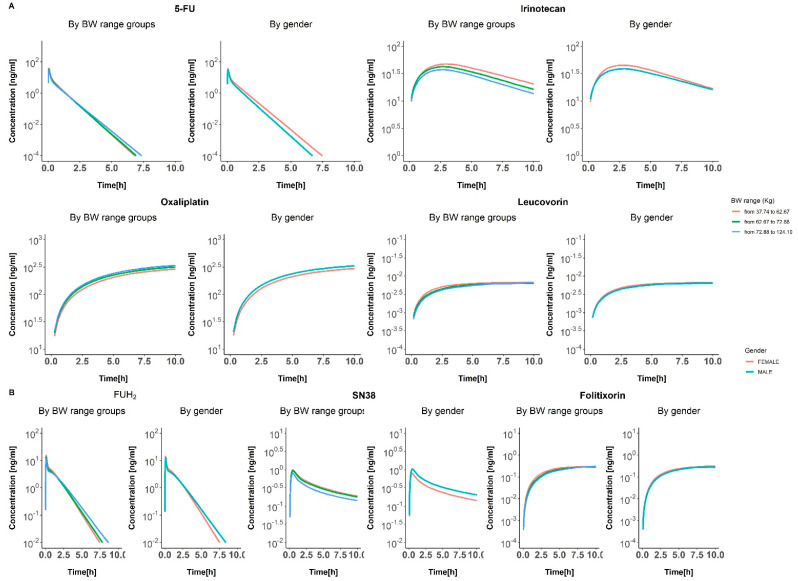
Colon concentration vs. time simulations for parent drugs (**A**) and metabolites (**B**). Solid lines indicate different conditions based on gender and body weight.

Figure 5 illustrates the impact of gender and body weight on the colon concentration–time profiles for the four drugs and their active metabolites. There are notable gender differences in the concentration profiles for 5-FU and irinotecan and their metabolites. For 5-FU, females generally exhibit higher concentrations in the colon compared to males, suggesting a gender-based difference in drug absorption or metabolism. A similar trend was observed for irinotecan, with males showing lower concentrations than females. In contrast, oxaliplatin displays higher concentrations in males, while leucovorin shows no gender-related differences.

Regarding body weight, irinotecan and its metabolite demonstrate the most significant differences. For the other drugs, body weight does not appear to significantly affect concentrations, except for a small difference observed for 5-FU and its metabolite.

## 4. Discussion

Given the high prevalence of colorectal cancer and its various forms in the colon, understanding the mechanisms of action of different treatment drugs is essential for optimizing patient care and improving therapy effectiveness. While classical pharmacokinetic models, which assume a central compartment is linked to peripheral compartments, are useful, they fall short in explaining the underlying mechanisms and drug metabolism in different organs. Physiologically-based pharmacokinetic (PBPK) models address these limitations by providing a comprehensive representation of drug behavior across body organs. This capability makes PBPK models ideal for visualizing drug distribution and customizing treatments based on individual patient characteristics, aiding in the selection of the optimal treatment approach.

During this exercise, four PBPK model were built using literature data. A total of 51 studies were compiled to contrast the observations with model predictions. This proves that published data is capable of building models without the needing to carry out more preclinical or clinical studies. This is an advantage because it may lead to a reduction in the number of clinical trials, thus reducing the number of people exposed to treatments that may not be effective, as well as a reduction in the high cost of these trials.

The four models were successfully developed using physicochemical parameters for each drug and were contrasted with the observations in plasma from the literature. Visual predictive checks are capable of capturing the variability of the observations mainly in leucovorin and 5-FU. For the case of the irinotecan and oxaliplatin, the area describing the range between the 5th and 95th percentiles does not capture some of the observations. This could be due to the larger number of observations included for irinotecan and oxaliplatin, resulting in greater data dispersion. Additionally, variability was initially included only in the age and weight of the population, as several PK models include these covariates. In a second step, variability was incorporated into the parameters governing the metabolism and elimination of the parent drugs, as their influence has been demonstrated in previous studies [67]. This approach represents a more realistic scenario, as there is natural variability in the clearance parameters of populations. As shown in Figure 3, this area expanded in all models, reflecting increased variability.

During a second step, active metabolites were included in the initial models to understand their behavior in the physiological structure. The typical model performance of the expanded models accurately captured the observations. The variability in observations was successfully described for FUH_2_ and folitixorin, whereas the SN38 model failed to capture all the data. This discrepancy could be attributed to the sparse data on SN38 sourced from the literature.

The precision of the models was examined by performing goodness-of-fit plots including a two-fold error range and a calculation of the AFE and AAFE, as regulatory guidelines such as the EMA and FDA recommend [68,69]. As was commented in the Results section, more than the 93% of the observations were found between the two-fold error range and more than 60% observations were found between the fold-error range, reflecting the high precision of the four models. In addition, the numerical evaluation reflects the robustness of these models, with the 87% seen in the AFE and AAFE indicators being acceptable and/or satisfactory. However, it is important to note that the oxaliplatin model presented with less robustness. This can be explained by the nature of the data, which represents a higher dispersion with respect to the other models. A refinement of the model may be needed to improve the accuracy of the model predictions.

Further exploration of the model was carried out in the colon, which is the target site of these four drugs. As is indicated above, there are no large differences between the colon segments regarding drug disposition, which make senses because the characteristics of the colon are similar along the segments as we can see with the values for flow rates and volumes in the different segments [70]. Interestingly, the colon/plasma AUC_inf_ ratio is similar for 5-FU, oxaliplatin, and leucovorin, but is significantly higher for irinotecan. This suggests that irinotecan accumulates more in the colon compared to the plasma. This fact is probably due to the high LogP value of irinotecan, which is 3.2, in contrast with the −0.89, −0.47, and −3.2 values of 5-FU, oxaliplatin, and leucovorin respectively. The higher LogP value indicates greater lipophilicity, which enhances the ability of irinotecan to penetrate cellular membranes. Additionally, in Table 4, the different partition coefficients for the rest of the body organs are shown, calculated using the PK-Sim standard method based on the Rodgers and Rowland approach. While it is true that other methods available in PK-Sim could have been tested, the lack of data for the colon has prevented us from verifying whether the selected method was the most appropriate, representing one of the limitations of our model. Our strategy was to use the PK-Sim standard model as a general approach for all drugs, ensuring consistency in the estimation of Kp values across all compounds and tissues. However, it is important to emphasize the value of PBPK models in providing data that cannot be obtained clinically, as they often require invasive procedures that may lack ethical justification.

In Table 4, it can be appreciated that irinotecan presents with a higher partition coefficient for all the organs, while the other three drugs are much lower. This supports the theory that was previously discussed, suggesting that lipophilicity is directly related to the biodistribution of the drug. Consequently, the fractional content of neutral lipids, phospholipids, and macromolecules is expected to be a key determinant of tissue drug partitioning [54,71]. This is significant because the characteristics of the tissue can influence the effect of the drug. Tumor tissues, however, differ from healthy tissues, and studies have shown that the Kp values of healthy and tumor tissues are often correlated. Using this basic model, we can predict the behavior of these drugs in tumors. In general, tumors with lower lipid and binding protein contents tend to exhibit lower Kp values, unless specific features of the tumor microenvironment favor the drug. For instance, 5-fluorouracil (5-FU) and leucovorin, being weak acids, may exhibit reduced Kp values in tumors due to the acidic interstitial pH, which results in lower ionization and reduced availability for cellular uptake. However, the high unbound fraction of 5-FU (Fu = 0.88) and its low logP (−0.89) suggest that it can distribute reasonably well in lipid-poor tumors. On the other hand, irinotecan, a weak base, may accumulate in tumors due to ionic trapping in the acidic environment. Finally, oxaliplatin, being a neutral compound, has its distribution primarily influenced by passive permeability and plasma protein binding.

Additionally, one of the strengths of the PBPK models developed in this study is their ability to assess the influence of both gender and body weight covariates at the target site.

With respect to body weight, irinotecan and its metabolite exhibit the most pronounced differences, reinforcing the previously reported influence of this covariate [72]. Individuals with a higher body weight tend to have lower concentrations of irinotecan and its metabolite in their colon. This highlights the importance of considering these covariates when optimizing treatment regimens for colorectal cancer patients, as differences in gender and body weight can significantly impact drug absorption, metabolism, and overall efficacy. By tailoring treatments based on these individual characteristics, healthcare providers can improve therapeutic outcomes, minimize adverse effects, and ensure that each patient receives the most effective and personalized care possible.

The next steps involve constructing a tumor compartment to explore its characteristics. It has been previously observed that tumor composition, including the contents of neutral lipids, phospholipids, and macromolecules, plays a critical role in determining drug distribution to the target site [54,71]. In this study, we have provided an initial assessment of drug distribution to the tumor based on its composition and the physicochemical properties of the drug. However, factors such as hypoxia and perfusion are also essential for accurately describing the biodistribution of the drug within the tumor [73]. As such, future steps will focus on incorporating these parameters into the model. Importantly, variability in these parameters among patients represents a key element for tailoring and optimizing treatment strategies, as differences in the tumor microenvironment can significantly influence the therapeutic efficacy and drug exposure. To achieve this, it is imperative to obtain drug concentration data within the tumor to validate the predictions of the model. However, acquiring such data poses ethical considerations, as invasive methods or costly imaging techniques are required. This underscores the necessity for models of this nature to both minimize costs and consider patient well-being. Nonetheless, a limitation arises as it is difficult to validate the accuracy of our tumor model. Despite this constraint, the model built in this study can be scaled for use in other species, such as mice, where drug concentrations within tumors have been measured. Consequently, it is feasible to establish the tumor compartment and verify the accuracy of our model. Therefore, our model serves as a foundational framework for expanding into tumor compartment modeling.

## Figures and Tables

**Table 1:** Physicochemical and disposition properties for parent drugs and metabolites.

	Irinotecan			5-FU			Oxaliplatin		Leucovorin		
	Parent Drug	Metabolite	Units	Parent Drug	Metabolite	Units	Parent Drug	Units	Parent Drug	Metabolite	Units
Physicochemical parameters											
Molecular weight	586.7 **	392.4 **	g/mol	130.08 **	114.1 **	g/mol	397.29 **	g/mol	473.446 *	459.5 *	g/mol
Lipophilicity	3.2 **	3.1.4 **	LogP	−0.89 **	−1.1 *	LogP	−0.47 *	LogP	−3.2 *	−0.5 *	LogP
Acid/Basic pKa	10.9 **	9.66 *	Dimensionless	8.02 **	11.73 *	Dimensionless	-	-	3.47 *	3.23 *	Dimensionless
Solubility at Ref-pH	0.107 **	0.29 **	mg/mL	5.86 **	25.9 **	mg/mL	27.5 *	mg/mL	0.297 *	0.33 *	mg/L
Disposition, metabolism and elimination parameters											
Fraction unbound	0.49 *	1 *	Dimensionless	0.88 [54]	1 **	Dimensionless	0.1 *	Dimensionless	0.85 *	0.44 *	Dimensionless
Renal Clearance	86.68 [55]	-	mL/min/Kg	2.38 [54]	-	mL/min/Kg	-	-	1.14 [56]	1.21 [56]	mL/min/Kg
Biliary Clearance	142.86 [55]	-	mL/min/Kg	-	-	-	-	-	-	-	-
Enzymatic Clearance	501.22 [55]	-	mL/min/Kg	-	-	-	-	-	297 [56]	-	mL/min
DPD Km	-	-	-	3.17 [54]	-	nmol/mL	-	-	-	-	-
DPD Vmax	-	-	-	22.5 [54]	-	ml/min/g tissue	-	-	-	-	-
Total Clearance	-	13.01 [57]	mL/min/Kg		1.18 [58]	L/h/Kg	2.5 [41]	L/h	-	-	-

5-FU, 5-fluorouracil; Ref-pH, Reference pH; DPD, dihydropyrimidine dehydrogenase; Km, Michaelos–Menten constant; Vmax, maximum rate constant. * Parameters obtained from DrugBank ** Parameters obtained from Pubchem.

**Table 2:** Numerical evaluation measurements, AFE and AAFE, for AUC_0-tend_, C_max_, and AUC_inf_, as calculated for each drug model.

	Irinotecan		5-FU		Oxaliplatin		Leucovorin	
	AFE	AAFE	AFE	AAFE	AFE	AAFE	AFE	AAFE
AUC_0-tend_	1.07	1.43	0.63	1.75	0.5	2	1.81	1.91
AUC_inf_	1.64	1.77	0.58	1.83	0.42	2.38	1.79	1.88
C_max_	1.52	1.53	0.97	2.15	0.5	1.96	0.56	1.77

5-FU, 5-fluorouracil; AUC_inf_, area under concentration vs. time curve from time of administration until drug is completely eliminated from the body; AUC_0-tend_, area under concentration–time curve at end of dosing interval; and C_max_, maximum concentration.

**Table 3:** The AUC_inf_ calculated for each drug in each part of the colon, and the vascular, interstitial, and intracellular spaces.

AUC_inf_ (µmol·Min/L)							
	Colon Ascendens						Blood
	Blood Cells	Plasma	Vascular	Interstitial	Intracellular	Total Colon Ascendens	Venous Plasma
Irinotecan	15.55	1.69	8.20	1.13	84.10	13.67	1.59
5-FU	6.16	10.36	8.39	9.23	5.01	8.53	9.17
Oxaliplatin	41.94	615.34	345.84	263.93	51.33	244.81	610.42
Leucovorin	13.39	17.67	15.65	15.42	16.40	15.58	18.20

5-FU, 5-fluorouracil; and AUC_inf_, the area under the concentration vs. time curve from the time of administration until the drug is completely eliminated from the body.

**Table 4:** Partition coefficients between intracellular space and plasma.

Partition Coefficients Between Intracellular Space and Plasma				
	Irinotecan	5-FU	Oxaliplatin	Leucovorin
Brain	90.78	0.74	0.06	0.7
Heart	84.21	0.68	0.08	0.64
Kidney	45.49	0.71	0.08	0.68
Stomach	52.9	0.72	0.08	0.69
Small intestine	52.9	0.72	0.08	0.69
Large intestine	52.9	0.72	0.08	0.69
Liver	59.45	0.69	0.08	0.66
Lung	11.77	0.74	0.08	0.71
Muscle	14.09	0.74	0.08	0.71
Pancreas	67.36	0.65	0.08	0.62
Spleen	16.81	0.71	0.08	0.69
Skin	86.3	0.59	0.07	0.56
Bone	221.66	0.48	0.09	0.43
Saliva	0.51	0.88	0.1	0.85

5-FU, 5-fluorouracil.

## Data Availability

The original contributions analyzed during the current study are available in the article/Supplementary Information. For any additional inquiries, kindly direct them to the corresponding author.

## Supplementary Materials

The following supporting information can be downloaded at the following link: https://www.mdpi.com/article/10.3390/pharmaceutics17010057/s1, Table S1: Clinical studies and patients characteristics. References [11,12,13,14,15,16,17,18,19,20,21,22,23,24,25,26,27,28,29,30,31,32,33,34,35,36,37,38,39,40,41,42,43,44,45,46,47,48,49,50,51,52,53] are cited in the Supplementary Materials
