# Supplementary material for: Development and Application of Physiologically-Based Pharmacokinetic Model to Predict Systemic and Organ Exposure of Colorectal Cancer Drugs
Source: Pharmaceutics. 2025 Jan 3;17(1):57. doi: 10.3390/pharmaceutics17010057 (PMC11768185; doi:10.3390/pharmaceutics17010057)
Supplement: Supplementary file 1 [file pharmaceutics-17-00057-s001.zip › Supplementary Table S1.pdf]

Suplementary Table S1. Clinical studies and patients characteristics.

Author	Molecule	Total number of patients	Sex (Male/female)	Median age (range)	CRC (%)	Dosing scheme
Atasilp 2018 [21]	Irinotecan/SN38	4			100	180 mg/m2 90-min infusion
Chabot 1995 [24]	Irinotecan	107	62/45	53 (29-74)	36	350/100 mg/m2 30-min IV infusion
Canal 1996 [22]	Irinotecan	47	26/21	(18-75)	100	350 mg/m2 30-min IV infusion
Oyaga-iriarte 1995 [23]	Irinotecan	46	22/24	56 (23-76)	26	100 mg/mL 30-min IV infusion
Corona 2008 [25]	Irinotecan/SN38	8	8/0	50 (32-68)	0	150 mg/m2 90-min infusion
Gbolahan 2021 [26]	Irinotecan/SN38	13	3/10	63 (36-72)	18	200 mg/m2 90-min infusion
M. Javle 2007 [27]	IIrinotecan/SN38	12	7/5		50	180 mg/m2 90-min infusion
Masuda 1994 [28]	Irinotecan/SN38	14	8/6	61 (43-74)	0	80/90 mg/m2 90-min infusion
Murren 2000 [29]	Irinotecan/SN38	21	15/6	54 (53-69)	0	50 mg/m2 90-min infusion
Rothenberg 1993 [30]	Irinotecan/SN38	32	_	55 (19-78)	68.75	125 mg/m2 90-min infusion
Suenaga 2014 [31]	Irinotecan	21	15/6	53.3 (32-72)	100	150 mg/m2 90-min infusion
Takahashi 1995 [32]	Irinotecan/SN38	9	8/1	67 (46-74)	100	60 mg/m2 90-min infusion
Wang 2016 [33]	Irinotecan/SN38	29	15/14	59 (53-75)	100	180 mg/m2 90-min infusion
Y Sasaki 1995 [34]	Irinotecan/SN38	12	7/5	56 (43-69)	16.7	100 mg/m2 90-min infusion
Y Sasaki 1995 [35]	IIrinotecan/SN38	36	28/8	60(29-75)	25	100 mg/m2 90-min infusion
Zhou 2004 [36]	Irinotecan/SN38	29	25/4	50 (27-71)	0	100 mg/m2 90-min infusion
Borner 2002 [11]	5-FU	17	13/14	58 (33-73)	100	400 mg/m2 IV bolus
Casale 2004 [12]	5-FU/FUH$_2$	18	11/7	58.5 (40-71)	100	400 mg/m2 IV bolus
Czejka 1993 [13]	5-FU	12	8/4	63.2 (49-70)	50	250 mg/m2 IV bolus
Di Paolo 2002 [14]	5-FU/FUH$_2$	80	48/32	61 (39-79)	100	370 mg/m2 IV bolus
Diasio 1989 [7]	5-FU	1*				500 mg/m2 IV bolus
Gusella 2005 [15]	5-FU	1*	1/0	64	0	325 mg/m2 IV bolus
Joel 2004 [16]	5-FU	9	8/1	68 (54-73)	0	400 mg/m2 IV bolus
Mc Dermott 1982 [8]	5-FU/FUH$_2$	12				10 mg/kg IV bolus
Saleem 2008 [17]	5-FU	8				380–407 mg/m2 IV bolus
Sugarmaker 2020 [18]	5-FU	1*	0/1	37	0	400 mg/m2 IV bolus
Woloch 2012 [19]	5-FU	127	81/46	60 (52-69)	100	370 mg/m2 IV bolus
Wright 2015 [20]	5-FU	23		9 (3-21)	0	500 mg/m2 IV bolus
Doroshow 2003 [37]	Oxaliplatin	12	28/32	62	47.7	130 mg/m2 2-h infusion
Gilmour 2003 [38]	Oxaliplatin	15				130 mg/m2 2-h infusion
Hea-Kyoung 2006 [39]	Oxaliplatin	9	5/4	62 (53-68)	100	130 mg/m2 2-h infusion
Kochi 2011 [40]	Oxaliplatin	5	3/2	58 (6.-65)	60	130 mg/m2 2-h infusion
Massari 1999 [41]	Oxaliplatin	13	7/6	62 (43-75)	30.1	130 mg/m2 2-h infusion
Perez-Ruixo 2013 [42]	Oxaliplatin	13	5/8	58.2		330 mg/m2 2-h infusion
Pieck 2008 [43]	Oxaliplatin	37	26/11	59 (32-80)	48	130 mg/m2 2-h infusion
Shirao 2006 [44]	Oxaliplatin	9	4/5	51(31-61)	44	130/90 mg/m2 2-h infusion
Takimoto 2013 [45]	Oxaliplatin	13	7/6	62	30.1	130 mg/m2 2-h infusion
Takimoto 2007 [46]	Oxaliplatin	34	23/11	64 (32-86)		130 mg/m2 2-h infusion
Van Cutsem 2007 [47]	Oxaliplatin	23	5/18	58 (46-71)	100	130 mg/m2 2-h infusion
Greiner et al. [48]	Leucovorin/Folitixorin	12	4/8	33	0	25 mg IV bolus
Machover 1986 [49]	Leucovorin/Folitixorin	25	13/12	62.5(42-82)	72	100 mg/m2 IV bolus
Schalhorn 1990 [50]	Leucovorin/Folitixorin	10	6/4	28 (25-33)	0	300 mg IV bolus

Schilsky 1990 [51]	Leucovorin/Folitixorin	5	1/4	28-35	0	1000 mg
Schleyer 2000 [52]	Leucovorin/Folitixorin	4	3/1	34 (31-38)	0	200 mg/m2 IV bolus
Trave 1988 [53]	Leucovorin/Folitixorin	12			100	500 mg/m2 2-h infusion
Zittoun 1993 [54]	Leucovorin/Folitixorin	14				200 mg/m2 IV bolus
